# Regularized gradient-projection methods for finding the minimum-norm solution of the constrained convex minimization problem

**DOI:** 10.1186/s13660-016-1289-4

**Published:** 2017-01-09

**Authors:** Ming Tian, Hui-Fang Zhang

**Affiliations:** 1 College of Science, Civil Aviation University of China, Tianjin, 300300 China; 2 Tianjin Key Laboratory for Advanced Signal Processing, Civil Aviation University of China, Tianjin, 300300 China

**Keywords:** 58E35, 47H09, 65J15, regularized gradient-projection method, minimum-norm, the constrained convex minimization problem, variational inequality

## Abstract

Let *H* be a real Hilbert space and *C* be a nonempty closed convex subset of *H*. Assume that *g* is a real-valued convex function and the gradient ∇*g* is $\frac{1}{L}$-ism with $L>0$. Let $0<\lambda <\frac{2}{L+2}$, $0<\beta_{n}<1$. We prove that the sequence $\{x_{n}\} $ generated by the iterative algorithm $x_{n+1}=P_{C}(I-\lambda(\nabla g+\beta_{n}I))x_{n}$, $\forall n\geq0$ converges strongly to $q\in U$, where $q=P_{U}(0)$ is the minimum-norm solution of the constrained convex minimization problem, which also solves the variational inequality $\langle-q, p-q\rangle\leq0$, $\forall p\in U$. Under suitable conditions, we obtain some strong convergence theorems. As an application, we apply our algorithm to solving the split feasibility problem in Hilbert spaces.

## Introduction

Let *H* be a real Hilbert space with inner product $\langle\cdot,\cdot \rangle$ and norm $\Vert \cdot \Vert $. Let *C* be a nonempty closed convex subset of *H*. Let $\mathbb{N}$ and $\mathbb{R}$ denote the sets of positive integers and real numbers. Suppose that *f* is a contraction on *H* with coefficient $0<\alpha<1$. A nonlinear operator $T:H\rightarrow H$ is nonexpansive if $\Vert Tx-Ty\Vert \leq \Vert x-y\Vert $ for all $x,y\in H$. We use $\operatorname {Fix}(T)$ to denote the fixed point of *T*.

Firstly, consider the constrained convex minimization problem:

1.1 $$ \min_{x\in C}g(x), $$

where $g:C\rightarrow\mathbb{R}$ is a real-valued convex function. Assume that the constrained convex minimization problem (1.1) is solvable, let *U* denote its solution set. The gradient-projection algorithm (GPA) is an effective method for solving the constrained convex minimization problem (1.1). A sequence $\{x_{n}\}$ generated by the following recursive formula:

1.2 $$ x_{n+1}=P_{C}(I-\lambda\nabla g)x_{n}, \quad \forall n \geq0, $$

where the parameter *λ* is real positive number. In general, if the gradient ∇*g* is *L*-Lipschitz continuous and *η*-strongly monotone, $0<\lambda<\frac{2\eta}{L^{2}}$, the sequence $\{x_{n}\}$ generated by (1.2) converges strongly to a minimizer of (1.1). However, if the gradient ∇*g* is only to be $\frac{1}{L}$-ism with $L>0$, $0<\lambda<\frac{2}{L}$, the sequence $\{x_{n}\}$ generated by (1.2) converges weakly to a minimizer of (1.1).

Recently, many authors combined the constrained convex minimization problem with a fixed point problem [1–3] and proposed composited iterative algorithms to find a solution of the constrained convex minimization problem [4–7].

In 2000, Moudafi [8] introduced the viscosity approximation method for nonexpansive mappings.

1.3 $$ x_{n+1}=\alpha_{n}f(x_{n})+(1- \alpha_{n})Tx_{n}, \quad \forall n\geq0. $$

In 2001, Yamada [9] introduced the so-called hybrid steepest-descent algorithm:

1.4 $$ x_{n+1}=Tx_{n}-\mu\lambda_{n}FTx_{n},\quad \forall n\geq0, $$

where *F* is Lipschitzian and strongly monotone operator. In 2006, Marino and Xu [10] considered a generative algorithm:

1.5 $$ x_{n+1}=\alpha_{n}\gamma f(x_{n})+(I- \alpha_{n}A)Tx_{n}, \quad \forall n\geq0, $$

where *A* is a strongly positive operator. In 2010, Tian [11] combined the iterative algorithm of (1.4), (1.5), and proposed a new iterative algorithm:

1.6 $$ x_{n+1}=\alpha_{n}\gamma f(x_{n})+(I-\mu \alpha_{n}F)Tx_{n}, \quad \forall n\geq0. $$

In 2010, Tian [12] generalized (1.6), obtained the following iterative algorithm:

1.7 $$ x_{n+1}=\alpha_{n}\gamma Vx_{n}+(I-\mu \alpha_{n}F)Tx_{n}, \quad \forall n\geq0, $$

where *V* is Lipschitzian operator. Based on these iterative algorithms, some authors combined GPA with averaged operator to solve the constrained convex minimization problem [13, 14].

In 2011, Ceng *et al.* [1] proposed a sequence $\{x_{n}\}$ generated by the following iterative algorithm:

1.8 $$ x_{n+1}=P_{C} \bigl[\theta_{n}rh(x_{n})+(I- \theta_{n}\mu F)T_{n}(x_{n}) \bigr],\quad \forall n \geq0, $$

where $h:C\rightarrow H$ is an *l*-Lipschitzian mapping with a constant $l>0$, and $F:C\rightarrow H$ is a *k*-Lipschitzian and *η*-strongly monotone operator with constants $k, \eta>0$. $\theta_{n}=\frac{2-\lambda_{n}L}{4}$, $P_{C}(I-\lambda_{n}\nabla g)=\theta_{n}I+(1-\theta_{n})T_{n}$, $\forall n\geq0$. Then a sequence $\{ x_{n}\}$ generated by (1.8) converges strongly to a minimizer of (1.1).

On the other hand, Xu [15] proposed that regularization can be used to find the minimum-norm solution of the minimization problem.

Consider the following regularized minimization problem:

$$ \min_{x\in C}g_{\beta}(x):=g(x)+\frac{\beta}{2}\Vert x \Vert ^{2}, $$

where the regularization parameter $\beta>0$. *g* is a convex function and the gradient ∇*g* is $\frac{1}{L}$-ism with $L>0$. Then the sequence $\{x_{n}\}$ generated by the following formula:

1.9 $$ x_{n+1}=P_{C}(I-\lambda\nabla g_{\beta_{n}})x_{n}=P_{C} \bigl(I-\lambda(\nabla g+\beta_{n}I) \bigr)x_{n}, \quad \forall n \geq0, $$

where the regularization parameters $0<\beta_{n}<1$, $0<\lambda<\frac {2}{L}$ converges weakly. But, if a sequence $\{x_{n}\}$ defined by

1.10 $$ x_{n+1}=P_{C}(I-\lambda_{n} \nabla g_{\beta_{n}})x_{n}=P_{C} \bigl(I-\lambda_{n}( \nabla g+\beta_{n}I) \bigr)x_{n},\quad \forall n\geq0, $$

where the initial guess $x_{0}\in C$, $\{\lambda_{n}\}$, $\{\beta_{n}\} $ satisfy the following conditions:

(i) $0<\lambda_{n}\leq\frac{\beta_{n}}{(L+\beta_{n})^{2}}$, $\forall n\geq 0$,
(ii) $\beta_{n}\rightarrow0$ (and $\lambda_{n}\rightarrow 0$) as $n\rightarrow\infty$,
(iii) $\sum_{n=1}^{\infty}\lambda_{n}\beta_{n} = \infty$,
(iv) $\frac{(\vert \lambda_{n}-\lambda_{n-1}\vert +\vert \lambda_{n}\beta_{n}-\lambda_{n-1}\beta_{n-1}\vert )}{(\lambda _{n}\beta_{n})^{2}}\rightarrow0$ as $n\rightarrow\infty$.

Then the sequence $\{x_{n}\}$ generated by (1.10) converges strongly to $x^{*}$, which is the minimum-norm solution of (1.1) [15].

Secondly, Yu et al. [16] proposed a strong convergence theorem with a regularized-like method to find an element of the set of solutions for a monotone inclusion problem in a Hilbert space.

### Theorem 1.1

[16]

*Let* *H* *be a real Hilbert space and* *C* *be a nonempty closed and convex subset of* *H*. *Let* $L>0$, *F* *is a* $\frac{1}{L}$-*ism mapping of* *C* *into* *H*. *Let* *B* *be a maximal monotone mapping on* *H* *and let* *G* *be a maximal monotone mapping on* *H* *such that the domains of* *B* *and* *G* *are included in* *C*. *Let* $J_{\rho}=(I+\rho B)^{-1}$ *and* $T_{r}=(I+rG)^{-1}$ *for each* $\rho>0$ *and* $r>0$. *Suppose that* $(F+B)^{-1}(0)\cap G^{-1}(0)\neq\emptyset$. *Let* $\{x_{n}\}\subset H$ *defined by*

1.11 $$ x_{n+1}=J_{\rho} \bigl(I-\rho(F+\beta_{n}I) \bigr)T_{r}x_{n}, \quad\forall n>0, $$

*where* $\rho\in(0,\infty)$, $\beta_{n}\in(0,1)$, $r\in(0,\infty)$. *Assume that*

(i) $0< a\leq\rho<\frac{2}{2+L}$,
(ii) $\lim_{n\rightarrow\infty}\beta_{n}=0$, $\sum_{n=1}^{\infty}\beta _{n}=\infty$.

*Then the sequence* $\{x_{n}\}$ *generated by* (1.11) *converges strongly to* *x̅*, *where* $\overline{x}= P_{(F+B)^{-1}(0)\cap G^{-1}(0)}(0)$.

From the article of Yu et al. [16], we obtain a new condition of parameter *ρ*, $0<\rho<\frac{2}{L+2}$, which is used widely in our article. Motivated and inspired by Lin, when $0<\lambda<\frac{2}{L+2}$, $\{\beta_{n}\}$ satisfy certain conditions, a sequence $\{x_{n}\}$ generated by the iterative algorithm (1.9):

$$ x_{n+1}=P_{C} \bigl(I-\lambda(\nabla g+\beta_{n}I) \bigr)x_{n}, \quad\forall n\geq0, $$

converges strongly to a point $q\in U$, where $q=P_{U}(0)$ is the minimum-norm solution of the constrained convex minimization problem.

Finally, we give concrete example and the numerical results to illustrate our algorithm is with fast convergence.

## Preliminaries

In this part, we introduce some lemmas that will be used in the rest part. Let *H* be a real Hilbert space and *C* be a nonempty closed convex subset of *H*. We use ‘→’ to denote strong convergence of the sequence $\{x_{n}\}$ and use ‘⇀’ to denote weak convergence.

Recall $P_{C}$ is the metric projection from *H* into *C*, then to each point $x\in H$, the unique point $P_{C}\in C$ satisfy the property:

$$ \Vert x-P_{C}x\Vert =\inf_{y\in C}\Vert x-y \Vert =: d(x,C). $$

$P_{C}$ has the following characteristics.

### Lemma 2.1

[17]

*For a given* $x\in H$:

1. $z=P_{C}x \Longleftrightarrow\langle x-z,z-y\rangle\geq0$, $\forall y\in C$;
2. $z=P_{C}x \Longleftrightarrow \Vert x-z\Vert ^{2}\leq \Vert x-y\Vert ^{2}-\Vert y-z\Vert ^{2}$, $\forall y\in C$;
3. $\langle P_{C}x-P_{C}y, x-y\rangle\geq \Vert P_{C}x-P_{C}y\Vert ^{2}$, $\forall x,y\in H$.

*From* (3), *we can derive that* $P_{C}$ *is nonexpansive and monotone*.

### Lemma 2.2

Demiclosed principle [18]

*Let* $T : C\rightarrow C$ *be a nonexpansive mapping with* $F(T)\neq\emptyset$. *If* $\{x_{n}\}$ *is a sequence in* *C* *weakly converging to* *x* *and if* $\{ (I-T)x_{n}\}$ *converges strongly to y*, *then* $(I-T)x = y$. *In particular*, *if* $y = 0$, *then* $x\in F(T)$.

### Lemma 2.3

[19]

*Let* $\{a_{n}\}$ *is a sequence of nonnegative real numbers such that*

$$a_{n+1} \leq(1-\alpha_{n})a_{n} + \alpha_{n}\delta_{n}, \quad n \geq0, $$

*where* $\{\alpha_{n}\}_{n=0}^{\infty}$ *and* $\{\delta_{n}\}_{n=0}^{\infty }$ *are sequences of real numbers in* $(0,1)$ *and such that*

(i) $\sum_{n=0}^{\infty}\alpha_{n} = \infty$;
(ii) $\limsup_{n\rightarrow\infty}\delta_{n} \leq0$ *or* $\sum_{n=0}^{\infty}\alpha_{n}\vert \delta_{n}\vert < \infty$.

*Then* $\lim_{n\rightarrow\infty}a_{n} = 0$.

## Main results

Let *H* be a real Hilbert space and *C* be a nonempty closed convex subset of *H*. Assume that $g:C\rightarrow\mathbb{R}$ is real-valued convex function and the gradient ∇*g* is $\frac{1}{L}$-ism with $L>0$. Suppose that the minimization problem (1.1) is consistent and let *U* denote its solution set. Let $0<\lambda<\frac{2}{L+2}$, $0<\beta _{n}<1$. Consider the following mapping $G_{n}$ on *C* defined by

$$ G_{n}x=P_{C} \bigl(I-\lambda(\nabla g+ \beta_{n}I) \bigr)x, \quad \forall x\in C, n\in\mathbb{N}. $$

We have

$$\begin{aligned} \Vert G_{n}x-G_{n}y\Vert ^{2} =& \bigl\Vert P_{C} \bigl(I-\lambda(\nabla g+\beta_{n}I) \bigr)x-P_{C} \bigl(I-\lambda(\nabla g+\beta_{n}I) \bigr)y \bigr\Vert ^{2} \\ \leq& \bigl\Vert \bigl(I-\lambda(\nabla g+\beta_{n}I) \bigr)x- \bigl(I-\lambda(\nabla g+\beta_{n}I) \bigr)y \bigr\Vert ^{2} \\ =&(1-\lambda\beta_{n})^{2}\Vert x-y\Vert ^{2}+\lambda^{2} \bigl\Vert \nabla g(x)-\nabla g(y) \bigr\Vert ^{2} \\ &{} -2\lambda(1-\lambda\beta_{n}) \bigl\langle x-y,\nabla g(x)- \nabla g(y) \bigr\rangle \\ \leq&(1-\lambda\beta_{n})^{2}\Vert x-y\Vert ^{2}+\lambda^{2} \bigl\Vert \nabla g(x)-\nabla g(y) \bigr\Vert ^{2} \\ &{} -\frac{2}{L}\lambda(1-\lambda\beta_{n}) \bigl\Vert \nabla g(x)-\nabla g(y) \bigr\Vert ^{2} \\ \leq&(1-\lambda\beta_{n})^{2}\Vert x-y\Vert ^{2}-\lambda \biggl(\frac{2}{L}(1-\lambda)-\lambda \biggr) \bigl\Vert \nabla g(x)-\nabla g(y) \bigr\Vert ^{2} \\ \leq&(1-\lambda\beta_{n})^{2}\Vert x-y\Vert ^{2}. \end{aligned}$$

That is,

$$ \Vert G_{n}x-G_{n}y\Vert \leq(1-\lambda \beta_{n})\Vert x-y\Vert . $$

Since $0<1-\lambda\beta_{n}<1$, it follows that $G_{n}$ is a contraction. Therefore, by the Banach contraction principle, $G_{n}$ has a unique fixed point $x_{n}$, such that

$$ x_{n}=P_{C} \bigl(I-\lambda(\nabla g+\beta_{n}I) \bigr)x_{n}. $$

Next, we prove that the sequence $\{x_{n}\}$ converges strongly to $q\in U$, which also solves the variational inequality

3.1 $$ \langle-q, p-q\rangle\leq0, \quad \forall p\in U. $$

Equivalently, $q=P_{U}(0)$, that is, *q* is the minimum-norm solution of the constrained convex minimization problem.

### Theorem 3.1

*Let* *C* *be a nonempty closed convex subset of a real Hilbert space* *H*. *Let* $g:C\rightarrow\mathbb{R}$ *is real*-*valued convex function and assume that the gradient* ∇*g* *is* $\frac{1}{L}$-*ism with* $L>0$. *Assume that* $U \neq\emptyset$. *Let* $\{x_{n}\}$ *be a sequence generated by*

3.2 $$ x_{n}=P_{C} \bigl(I-\lambda(\nabla g+\beta_{n}I) \bigr)x_{n}, \quad\forall n\in\mathbb{N}. $$

*Let* *λ*, $\{\beta_{n}\}$ *satisfy the following conditions*:

(i) $0<\lambda<\frac{2}{2+L}$,
(ii) $\{\beta_{n}\}\subset(0,1)$, $\lim_{n\rightarrow\infty}\beta _{n}=0$, $\sum_{n=1}^{\infty}\beta_{n} = \infty$.

*Then* $\{x_{n}\}$ *converges strongly to a point* $q\in U$, *where* $q=P_{U}(0)$, *which is the minimum*-*norm solution of the minimization problem* (1.1) *and also solves the variational inequality* (3.1).

### Proof

First, we claim that $\{x_{n}\}$ is bounded. Indeed, pick any $p\in U$, then we have

$$\begin{aligned} \Vert x_{n}-p\Vert =& \bigl\Vert P_{C} \bigl(I- \lambda(\nabla g+\beta_{n}I) \bigr)x_{n}-P_{C}(I- \lambda\nabla g)p \bigr\Vert \\ \leq& \bigl\Vert \bigl(I-\lambda(\nabla g+\beta_{n}I) \bigr)x_{n}- \bigl(I-\lambda(\nabla g+\beta_{n}I) \bigr)p \bigr\Vert \\ &{}+ \bigl\Vert \bigl(I-\lambda(\nabla g+\beta_{n}I) \bigr)p-(I- \lambda\nabla g)p \bigr\Vert \\ \leq&(1-\lambda\beta_{n})\Vert x_{n}-p\Vert +\lambda \beta_{n}\Vert p\Vert . \end{aligned}$$

Then we derive that

$$ \Vert x_{n}-p\Vert \leq \Vert p\Vert , $$

and hence $\{x_{n}\}$ is bounded.

Next, we claim that $\Vert x_{n}-P_{C}(I-\lambda\nabla g )x_{n}\Vert \rightarrow0$. Indeed

$$\begin{aligned} \bigl\Vert x_{n}-P_{C}(I-\lambda\nabla g)x_{n} \bigr\Vert =& \bigl\Vert P_{C} \bigl(I-\lambda( \nabla g+\beta_{n}I) \bigr)x_{n}-P_{C}(I-\lambda \nabla g)x_{n} \bigr\Vert \\ \leq& \bigl\Vert \bigl(I-\lambda(\nabla g+\beta_{n}I) \bigr)x_{n}-(I-\lambda\nabla g)x_{n} \bigr\Vert \\ \leq&\lambda\beta_{n}\Vert x_{n}\Vert . \end{aligned}$$

Since $\{x_{n}\}$ is bounded, $\beta_{n}\rightarrow0$ ($n\rightarrow \infty$), we obtain

$$ \bigl\Vert x_{n}-P_{C}(I-\lambda\nabla g)x_{n} \bigr\Vert \rightarrow0. $$

∇*g* is $\frac{1}{L}$-ism. Consequently, $P_{C}(I-\lambda\nabla g)$ is a nonexpansive self-mapping on *C*. As a matter of fact, we have for each $x,y\in C$

$$\begin{aligned}& \bigl\Vert P_{C}(I-\lambda\nabla g)x-P_{C}(I-\lambda \nabla g)y \bigr\Vert ^{2} \\& \quad \leq \bigl\Vert (I-\lambda\nabla g)x-(I- \lambda\nabla g)y \bigr\Vert ^{2} \\& \quad = \bigl\Vert x-y-\lambda \bigl(\nabla g(x)-\nabla g(y) \bigr) \bigr\Vert ^{2} \\& \quad = \Vert x-y\Vert ^{2}-2\lambda \bigl\langle x-y, \nabla g(x)- \nabla g(y) \bigr\rangle +\lambda^{2} \bigl\Vert \nabla g(x)-\nabla g(y) \bigr\Vert ^{2} \\& \quad \leq \Vert x-y\Vert ^{2}-\lambda \biggl(\frac{2}{L}-\lambda \biggr) \bigl\Vert \nabla g(x)-\nabla g(y) \bigr\Vert ^{2} \\& \quad \leq \Vert x-y\Vert ^{2}. \end{aligned}$$

$\{x_{n}\}$ is bounded, consider a subsequence $\{x_{n_{i}}\}$ of $\{ x_{n}\}$. Since $\{x_{n_{i}}\}$ is bounded, there exists a subsequence $\{x_{n_{i_{j}}}\}$ of $\{x_{n_{i}}\}$ which converges weakly to *z*. Without loss of generality, we can assume that $x_{n_{i}}\rightharpoonup z$. Then by Lemma 2.2, we obtain $z\in U$.

On the other hand

$$\begin{aligned} \Vert x_{n}-z\Vert ^{2} =& \bigl\Vert P_{C} \bigl(I-\lambda(\nabla g+\beta_{n}I) \bigr)x_{n}-P_{C}(I-\lambda\nabla g)z \bigr\Vert ^{2} \\ \leq& \bigl\langle \bigl(I-\lambda(\nabla g+\beta_{n}I) \bigr)x_{n}-(I-\lambda\nabla g)z, x_{n}-z \bigr\rangle \\ =& \bigl\langle \bigl(I-\lambda(\nabla g+\beta_{n}I) \bigr)x_{n}- \bigl(I-\lambda(\nabla g+\beta_{n}I) \bigr)z, x_{n}-z \bigr\rangle \\ &{} +\langle-\lambda\beta_{n}z, x_{n}-z\rangle \\ \leq&(1-\lambda\beta_{n})\Vert x_{n}-z\Vert ^{2}+\lambda\beta_{n}\langle-z, x_{n}-z\rangle. \end{aligned}$$

Thus

$$\Vert x_{n}-z\Vert ^{2}\leq\langle-z, x_{n}-z\rangle. $$

In particular

$$\Vert x_{n_{i}}-z\Vert ^{2}\leq\langle-z, x_{n_{i}}-z\rangle. $$

Since $x_{n_{i}}\rightharpoonup z$. Then we derive that $x_{n_{i}}\rightarrow z$ as $i\rightarrow\infty$.

Let *q* be the minimum-norm solution of *U*, that is, $q=P_{U}(0)$. Since $\{x_{n}\}$ is bounded, there exists a subsequence $\{x_{n_{i}}\} $ of $\{x_{n}\}$ such that $x_{n_{i}}\rightharpoonup z$. As the above proof, we know that $x_{n_{i}}\rightarrow z$, $z\in U$.

Then we derive that

$$\begin{aligned} \Vert x_{n}-q\Vert ^{2} =& \bigl\Vert P_{C} \bigl(I-\lambda(\nabla g+\beta_{n}I) \bigr)x_{n}-q \bigr\Vert ^{2} \\ \leq& \bigl\langle \bigl(I-\lambda(\nabla g+\beta_{n}I) \bigr)x_{n}-(I-\lambda\nabla g)q, x_{n}-q \bigr\rangle \\ =& \bigl\langle \bigl(I-\lambda(\nabla g+\beta_{n}I) \bigr)x_{n}- \bigl(I-\lambda(\nabla g+\beta_{n}I) \bigr)q, x_{n}-q \bigr\rangle \\ &{}+\langle-\lambda\beta_{n}q, x_{n}-q\rangle \\ \leq&(1-\lambda\beta_{n})\Vert x_{n}-q\Vert ^{2}+\lambda\beta_{n}\langle-q, x_{n}-q\rangle. \end{aligned}$$

Thus

$$\Vert x_{n}-q\Vert ^{2}\leq\langle-q, x_{n}-q\rangle. $$

In particular

$$\Vert x_{n_{i}}-q\Vert ^{2}\leq\langle-q, x_{n_{i}}-q\rangle. $$

Since $x_{n_{i}}\rightarrow z$, $z\in U$,

$$\Vert z-q\Vert ^{2}\leq\langle-q, z-q\rangle\leq0. $$

So, we have $z=q$. From the arbitrariness of $z\in U$, it follows that $q\in U$ is a solution of the variational inequality (3.1). By the uniqueness of solution of the variational inequality (3.1), we conclude that $x_{n}\rightarrow q$ as $n\rightarrow\infty$, where $q=P_{U}(0)$. □

### Theorem 3.2

*Let* *C* *be a nonempty closed convex subset of a real Hilbert space* *H* *and* $g:C\rightarrow\mathbb{R}$ *is real*-*valued convex function and assume that the gradient* ∇*g* *is* $\frac{1}{L}$-*ism with* $L>0$. *Assume that* $U\neq\emptyset$. *Let* $\{x_{n}\}$ *be a sequence generated by* $x_{1}\in C$ *and*

3.3 $$ x_{n+1}=P_{C} \bigl(I-\lambda(\nabla g+\beta_{n}I) \bigr)x_{n}, \quad\forall n\in\mathbb{N}, $$

*where* *λ* *and* $\{\beta_{n}\}$ *satisfy the following conditions*:

(i) $0<\lambda<\frac{2}{L+2}$;
(ii) $\{\beta_{n}\}\subset(0,1)$, $\lim_{n\rightarrow\infty}\beta _{n}=0$, $\sum_{n=1}^{\infty}\beta_{n} = \infty$, $\sum_{n=1}^{\infty }\vert \beta_{n+1}-\beta_{n}\vert <\infty$.

*Then* $\{x_{n}\}$ *converges strongly to a point* $q\in U$, *where* $q=P_{U}(0)$, *which is the minimum*-*norm solution of the minimization problem* (1.1) *and also solves the variational inequality* (3.1).

### Proof

First, we claim that $\{x_{n}\}$ is bounded. Indeed, pick any $p\in U$, then we know that, for any $n\in\mathbb{N}$,

$$\begin{aligned} \Vert x_{n+1}-p\Vert \leq& \bigl\Vert P_{C} \bigl(I- \lambda(\nabla g+\beta_{n}I) \bigr)x_{n}-P_{C} \bigl(I-\lambda(\nabla g+\beta_{n}I) \bigr)p \bigr\Vert \\ &{}+ \bigl\Vert P_{C} \bigl(I-\lambda(\nabla g+ \beta_{n}I) \bigr)p-P_{C}(I-\lambda\nabla g)p \bigr\Vert \\ \leq&(1-\lambda\beta_{n})\Vert x_{n}-p\Vert +\lambda \beta_{n}\Vert p\Vert \\ \leq&\max \bigl\{ \Vert x_{n}-p\Vert ,\Vert p\Vert \bigr\} . \end{aligned}$$

By the introduction

$$\begin{aligned} \Vert x_{n}-p\Vert \leq \max \bigl\{ \Vert x_{1}-p \Vert ,\Vert p\Vert \bigr\} , \end{aligned}$$

and hence $\{x_{n}\}$ is bounded.

Next, we show that $\Vert x_{n+1}-x_{n}\Vert \rightarrow0$.

$$\begin{aligned} \Vert x_{n+1}-x_{n}\Vert =& \bigl\Vert P_{C} \bigl(I-\lambda(\nabla g+\beta_{n}I) \bigr)x_{n}-P_{C} \bigl(I-\lambda(\nabla g+ \beta_{n-1}I) \bigr)x_{n-1} \bigr\Vert \\ \leq& \bigl\Vert \bigl(I-\lambda(\nabla g+\beta_{n}I) \bigr)x_{n}- \bigl(I-\lambda(\nabla g+\beta_{n-1}I) \bigr)x_{n-1} \bigr\Vert \\ =& \bigl\Vert \bigl(I-\lambda(\nabla g+\beta_{n}I) \bigr)x_{n}- \bigl(I-\lambda(\nabla g+\beta_{n}I) \bigr)x_{n-1} \\ &{} -\lambda\beta_{n}x_{n-1}+ \lambda\beta_{n-1}x_{n-1} \bigr\Vert \\ \leq&(1-\lambda\beta_{n})\Vert x_{n}-x_{n-1} \Vert +\lambda \vert \beta_{n}-\beta_{n-1}\vert \cdot \Vert x_{n-1}\Vert \\ \leq&(1-\lambda\beta_{n})\Vert x_{n}-x_{n-1} \Vert +\lambda \vert \beta_{n}-\beta_{n-1}\vert \cdot M, \end{aligned}$$

where $M=\sup\{\Vert x_{n}\Vert :n\in\mathbb{N}\}$. Hence, by Lemma 2.3, we have

$$\Vert x_{n+1}-x_{n}\Vert \rightarrow0. $$

Then we claim that $\Vert x_{n}-P_{C}(I-\lambda\nabla g)x_{n}\Vert \rightarrow0$.

$$\begin{aligned} \bigl\Vert x_{n}-P_{C}(I-\lambda\nabla g)x_{n} \bigr\Vert =& \bigl\Vert x_{n}-x_{n+1}+x_{n+1}-P_{C}(I- \lambda\nabla g)x_{n} \bigr\Vert \\ \leq&\Vert x_{n}-x_{n+1}\Vert + \bigl\Vert P_{C} \bigl(I-\lambda(\nabla g+\beta_{n}I) \bigr)x_{n}-P_{C}(I-\lambda\nabla g)x_{n} \bigr\Vert \\ \leq&\Vert x_{n}-x_{n+1}\Vert +\lambda \beta_{n}\cdot \Vert x_{n}\Vert \\ \leq&\Vert x_{n}-x_{n+1}\Vert +\lambda \beta_{n}\cdot M, \end{aligned}$$

since $\beta_{n}\rightarrow0$ and $\Vert x_{n+1}-x_{n}\Vert \rightarrow0$, we have

$$\begin{aligned} \bigl\Vert x_{n}-P_{C}(I-\lambda\nabla g)x_{n} \bigr\Vert \rightarrow0. \end{aligned}$$

Next, we show that

3.4 $$\begin{aligned} \limsup_{n\rightarrow\infty}\langle-q, x_{n}-q\rangle\leq0. \end{aligned}$$

Let *q* be the minimum-norm solution of *U*, that is, $q=P_{U}(0)$. Since $\{x_{n}\}$ is bounded, without loss of generality, we assume that $x_{n_{j}}\rightharpoonup z$. By the same argument as in the proof of Theorem 3.1, we have $z\in U$.

$$\begin{aligned} \limsup_{n\rightarrow\infty}\langle-q, x_{n}-q\rangle=\lim _{j\rightarrow\infty}\langle-q, x_{n_{j}}-q\rangle=\langle-q, z-q \rangle\leq0. \end{aligned}$$

Then

$$\begin{aligned} \Vert x_{n+1}-q\Vert ^{2} =& \bigl\Vert P_{C} \bigl(I-\lambda(\nabla g+\beta_{n}I) \bigr)x_{n}-P_{C}(I-\lambda\nabla g)q \bigr\Vert ^{2} \\ =& \bigl\langle P_{C} \bigl(I-\lambda(\nabla g+\beta_{n}I) \bigr)x_{n}-P_{C} \bigl(I-\lambda(\nabla g+ \beta_{n}I) \bigr)q, x_{n+1}-q \bigr\rangle \\ &{}+ \bigl\langle P_{C} \bigl(I-\lambda(\nabla g+ \beta_{n}I) \bigr)q-P_{C}(I-\lambda\nabla g)q, x_{n+1}-q \bigr\rangle \\ \leq&(1-\lambda\beta_{n})\Vert x_{n}-q\Vert \cdot \Vert x_{n+1}-q\Vert +\lambda\beta_{n}\langle-q, x_{n+1}-q\rangle \\ \leq&\frac{1-\lambda\beta_{n}}{2}\Vert x_{n}-q\Vert ^{2}+ \frac{1}{2}\Vert x_{n+1}-q\Vert ^{2}+\lambda \beta_{n}\langle-q, x_{n+1}-q\rangle. \end{aligned}$$

It follows that

$$\begin{aligned} \Vert x_{n+1}-q\Vert ^{2} \leq&(1-\lambda \beta_{n})\Vert x_{n}-q\Vert ^{2}+2\lambda \beta_{n}\langle-q, x_{n+1}-q\rangle \\ =&(1-\lambda\beta_{n})\Vert x_{n}-q\Vert ^{2}+2\lambda\beta_{n}\delta_{n}, \end{aligned}$$

where $\delta_{n}=\langle-q, x_{n+1}-q\rangle$.

It is easy to see that $\lim_{n\rightarrow\infty}\lambda\beta_{n}=0$, $\sum_{n=1}^{\infty}\lambda\beta_{n} = \infty$ and $\limsup_{n\rightarrow \infty}\delta_{n}\leq0$. Hence, by Lemma 2.3, the sequence $\{x_{n}\}$ converges strongly to *q*, where $q=P_{U}(0)$. This completes the proof. □

## Application

In this part, we will illustrate the practical value of our algorithm in the split feasibility problem. In 1994, Censor and Elfving [20] came up with the split feasibility problem. The SFP is formulated as finding a point *x* with the property:

4.1 $$ x\in C \quad \mbox{and} \quad Ax\in Q, $$

where *C* and *Q* are nonempty closed and convex subset of real Hilbert spaces $H_{1}$ and $H_{2}$, $A:H_{1}\rightarrow H_{2}$ is bounded linear operator.

Next, we consider the constrained convex minimization problem:

4.2 $$ \min_{x\in C}g(x)=\min_{x\in C}\frac{1}{2} \Vert Ax-P_{Q}Ax\Vert ^{2}. $$

If $x^{*}$ is a solution of SFP, then $Ax^{*}\in Q$ and $Ax^{*}-P_{Q}Ax^{*}=0$, $x^{*}$ is the solution of the minimization problem (4.2). The gradient of *g* is ∇*g*, where $\nabla g=A^{*}(I-P_{Q})A$. Applying Theorem 3.2, we obtain the following theorem.

### Theorem 4.1

*Assume that the SFP* (4.1) *is consistent*. *Let* *C* *be a nonempty closed convex subset of a real Hilbert space* *H*. *Assume that* $A:H_{1}\rightarrow H_{2}$ *is bounded linear operator*, $W\neq\emptyset $, *where* *W* *denotes the solution set of SFP* (4.1). *Let* $\{x_{n}\}$ *be a sequence generated by* $x_{1}\in C$ *and*

4.3 $$ x_{n+1}=P_{C} \bigl(I-\lambda \bigl(A^{*}(I-P_{Q})A+ \beta_{n}I \bigr) \bigr)x_{n}, \quad\forall n\in\mathbb{N}. $$

*Let* *λ* *and* $\{\beta_{n}\}$ *satisfy the following conditions*:

(i) $0<\lambda<\frac{2}{2+\Vert A\Vert ^{2}}$;
(ii) $\{\beta_{n}\}\subset(0,1)$, $\lim_{n\rightarrow\infty}\beta _{n}=0$, $\sum_{n=1}^{\infty}\beta_{n} = \infty$, $\sum_{n=1}^{\infty }\vert \beta_{n+1}-\beta_{n}\vert <\infty$.

*Then* $\{x_{n}\}$ *converges strongly to a point* $q\in W$, *where* $q=P_{W}(0)$.

### Proof

We only need to show that ∇*g* is $\frac{1}{\Vert A\Vert ^{2}}$-ism, then Theorem 4.1 can be obtained by Theorem 3.2.

$$\begin{aligned} \nabla g=A^{*}(I-P_{Q})A. \end{aligned}$$

Since $P_{Q}$ is firmly nonexpansive, so $P_{Q}$ is $\frac {1}{2}$-averaged mapping, then $I-P_{Q}$ is 1-ism, for any $x,y\in C$, we derive that

$$\begin{aligned} \bigl\langle \nabla g(x)-\nabla g(y), x-y \bigr\rangle =& \bigl\langle A^{*}(I-P_{Q})Ax-A^{*}(I-P_{Q})Ay, x-y \bigr\rangle \\ =& \bigl\langle (I-P_{Q})Ax-(I-P_{Q})Ay, Ax-Ay \bigr\rangle \\ \geq& \bigl\Vert (I-P_{Q})Ax-(I-P_{Q})Ay \bigr\Vert ^{2} \\ =&\frac{1}{\Vert A\Vert ^{2}}\cdot \bigl\Vert A^{*} \bigl((I-P_{Q})Ax-(I-P_{Q})Ay \bigr) \bigr\Vert ^{2} \\ =&\frac{1}{\Vert A\Vert ^{2}}\cdot \bigl\Vert \nabla g(x)-\nabla g(y) \bigr\Vert ^{2}. \end{aligned}$$

So, ∇*g* is $\frac{1}{\Vert A\Vert ^{2}}$-ism. □

## Numerical result

In this part, we use the algorithm in Theorem 4.1 to solve a system of linear equations. Then we calculate the $4\times4$ system of linear equations.

### Example 1

Let $H_{1}=H_{2}=\mathbb{R}^{4}$. Take

5.1 $$\begin{aligned}& A=\left ( \textstyle\begin{array}{c@{\quad}c@{\quad}c@{\quad}c} 1&-1&2&-1\\ 2&-2&3&-3\\ 1&1&1&0\\ 1&-1&4&3 \end{array}\displaystyle \right ), \end{aligned}$$

5.2 $$\begin{aligned}& b=\left ( \textstyle\begin{array}{c} -2\\ -10\\ 6\\ 18 \end{array}\displaystyle \right ). \end{aligned}$$

Then the SFP can be formulated as the problem of finding a point $x^{*}$ with the property

$$x^{*}\in C \quad \mbox{and} \quad Ax^{*}\in Q, $$

where $C=\mathbb{R}^{4}$, $Q=\{b\}$. That is, $x^{*}$ is the solution of the system of linear equations $Ax=b$, and

5.3 $$ x^{*}=\left ( \textstyle\begin{array}{c} 1\\ 3\\ 2\\ 4 \end{array}\displaystyle \right ).$$

Take $P_{C}=I$, where *I* denotes the $4\times4$ identity matrix. Given the parameters $\beta_{n}=\frac{1}{(n+2)^{2}}$ for $n\geq0$, $\lambda =\frac{3}{200}$. Then by Theorem 4.1, the sequence $\{x_{n}\}$ is generated by

$$x_{n+1} =x_{n}-\frac{3}{200}A^{*}Ax_{n}+ \frac{3}{200}A^{*}b-\frac{3}{200(n+2)^{2}}x_{n}. $$

As $n\rightarrow\infty$, we have $\{x_{n}\}\rightarrow x^{*}=(1,3,2,4)^{T}$.

From Table 1, we can easily see that with iterative number increasing $x_{n}$ approaches to the exact solution $x^{*}$ and the errors gradually approach zero.

**Table 1:** **Numerical results as regards Example** **1**

***n***	$\boldsymbol{x_{n}^{1}}$	$\boldsymbol{x_{n}^{2}}$	$\boldsymbol{x_{n}^{3}}$	$\boldsymbol{x_{n}^{4}}$	$\boldsymbol{E_{n}}$
0	1.0000	1.0000	1.0000	1.0000	5.74E + 00
100	1.2292	2.8506	1.8424	4.0887	3.28E − 01
1,000	1.2208	2.9107	1.8691	4.0722	2.81E − 01
5,000	1.1128	2.9543	1.9331	4.0369	1.42E − 01
10,000	1.0298	2.9880	1.9824	4.0097	3.79E − 02

In Tian and Jiao [21], they use another iterative algorithm to calculate the same example.

Compare Table 1 with Table 2, we find that if the parameters $\beta _{n}$ are the same, when $\lambda\rightarrow\frac{2}{L+2}$, our algorithm is with fast convergence.

**Table 2:** **Numerical results as regards Example** **1**

***n***	$\boldsymbol{x_{n}^{1}}$	$\boldsymbol{x_{n}^{2}}$	$\boldsymbol{x_{n}^{3}}$	$\boldsymbol{x_{n}^{4}}$	$\boldsymbol{E_{n}}$
0	1.0000	1.0000	1.0000	1.0000	3.74E + 00
100	0.6070	2.0706	1.7816	3.9672	1.03E + 00
1,000	1.0094	2.8884	1.9496	4.0123	1.23E − 01
5,000	1.0353	2.9643	1.9702	4.0133	5.99E − 02
10,000	1.0307	2.9769	1.9774	4.0109	4.59E − 02

## Conclusion

In a real Hilbert space, there are many methods to solve the constrained convex minimization problem. However, most of them cannot find the minimum-norm solution. In this article, we use the regularized gradient-projection algorithm to find the minimum-norm solution of the constrained convex minimization problem, where $0<\lambda<\frac {2}{L+2}$. Then under some suitable conditions, new strong convergence theorems are obtained. Finally, we apply this algorithm to the split feasibility problem and use a concrete example and numerical results to illustrate that our algorithm has fast convergence.
